# Genome-wide association study and genomic heritabilities for blood protein levels in Lori-Bakhtiari sheep

**DOI:** 10.1038/s41598-021-03290-z

**Published:** 2021-12-09

**Authors:** P. Zamani, H. Mohammadi, S. Z. Mirhoseini

**Affiliations:** 1 Department of Animal Science, Faculty of Agriculture, Bu-Ali Sina University, Hamedan, Iran; 2 Department of Animal Science, Faculty of Agriculture, University of Guilan, Rasht, Iran

**Keywords:** Genome, Genome-wide association studies

## Abstract

Serum protein levels are related to physiological and pathological status of animals and could be affected by both genetic and environmental factors. This study aimed to evaluate genetic variation of serum protein profile in sheep. Blood samples were randomly collected from 96 Lori-Bakhtiari ewes, a heavy meat-type breed. Total protein, albumin, globulin, α1, α2, β and γ globulins and IgG levels were measured in blood serum. The samples were genotyped using the Illumina OvineSNP50 BeadChip. The studied traits adjusted for age, birth type, birth season and estimate of breeding value for body weight were considered as pseudo-phenotypes in genome-wide association analysis. In the GWAS model, the first five principal components were fitted as covariates to correct the biases due to possible population stratification. The Plink, R and GCTA software were used for genome-wide association analysis, construction of Q-Q and Manhattan plots and estimation of genetic variances, respectively. Noticeable genomic heritabilities ± SE were estimated for total and γ globulins (0.868 ± 0.262 and 0.831 ± 0.364, respectively), but other protein fractions had zero or close to zero estimates. Based on the Bonferroni adjusted *p* values, four QTLs located on 181.7 Mbp of OAR3, 107.7 Mbp of OAR4, 86.3 Mbp of OAR7 and 83.0 Mbp of OAR8 were significantly associated with α1, β, β and γ globulins, respectively. The results showed that the *PKP2*, *IGF2R*, *SLC22A1* and *SLC22A2* genes could be considered as candidate genes for blood serum proteins. The present study showed significant genetic variations of some blood protein fractions.

## Introduction

Animal health and physiological status are main concerns for all livestock producers. Whereby, any physiological and disease problem may endanger livestock production enterprises. Blood serum protein levels could be considered as important indicators for physiological status of animals and a diagnostic aid for many disease types and health disorders^1,2^.

Several functions could be stated for blood serum proteins. For example, albumin involves in binding and transportation of many substances^3^. Globulins, including α, β and γ-globulins, constitute a major category of serum proteins, which include antibodies and involve in hemostatic-fibrinolytic pathways, and transportation of several important molecules, such as Iron, hormones, lipids and vitamins^4^.

It is proven that ovine blood serum protein profile could be affected by several factors, such as age^2,5–7^, sex^5,7^, body condition score^8^, health condition^9^, season^10,11^, gestation stage^12^ and birth season^6^. Blood serum protein profile also varies among different ruminant species^13^ and ovine breeds^10,14,15^.

Low to moderate heritabilities have been estimated for serum total protein (0.20), albumin (0.13), globulins (0.20) and albumin/globulins ratio (0.21) in an Italian Holstein cattle population^16^. There are some reports on genetic diversities of blood serum albumin^17–19^ and albumin/globulins ratio^20^, but genetic variations of other fractions, including, α, β and γ globulins are mostly unknown.

It has been found that net benefits of selection for disease resistance, would outweigh the opportunity cost of reduced genetic progress in other traits^21^. Detection of QTLs and candidate genes associated with blood serum fractions is a potential method to design an appropriate marker assisted selection program to improve animal health situation and resistance to environmental conditions. A few genome-wide association studies (GWAS) on blood serum protein levels in human, mostly on total protein, albumin and albumin/globulin ratio^20,22–24^. No GWAS report on blood serum protein levels in animals, especially sheep was found in literature. The aim of this research was to conduct a genome-wide association study, to identify possible QTLs and candidate genes associated with blood serum protein fractions in sheep.

## Methods

### Population and samples

This study was conducted on Lori-Bakhtiari sheep, a heavy meat-type breed of sheep in Zagros area, where is known as the first center of sheep domestication^25^. The studied population was a research flock in Shooli Sheep Breeding Station (32.31362° N, 51.05340° E), Chaharmahal va Bakhtiari province, Iran. In the studied population, the animals regularly graze on pasture or field residuals during spring to mid-autumn and in other times are kept indoors and fed by the diets, mainly composed by alfalfa, wheat or barley straw and barley grain. The mating season begins in late August to elongates to late October. More detailed information about climate, diets and managerial conditions in the studied population are provided by Almasi et al.^26^.

A total of 96 ewes were randomly selected among 450 available ewes, in September. The health status of the selected animals was evaluated, based on appetite, body temperature, fecal consistency and parasite tests. Blood samples were taken from jugular vein and collected in both EDTA-free and EDTA-containing tubes.

### Blood serum parameters

The samples, collected in EDTA-free tubes, were centrifuged at 2100*g* for 5 min to separate the serum fraction, which was then used to measure a variety of blood serum protein fractions. The measured serum proteins, were total protein, albumin, total globulins, immunoglobulin G (IgG), and α1, α2, β and γ globulins. Total protein was measured by Biuret method^27^, using a Biochemistry Auto Analyzer (Sinnowa D280, China). Different protein fractions, including, albumin, total globulins and α1, α2, β and γ globulins were separated by electrophoresis of the samples on cellulose acetate strips (Helena Biosciences, UK) in 180 V for 20 min. Helena electrophoresis interpretation software (Helena Biosciences, UK) was used to read the bands. The IgG levels were measured by the enzyme-linked immunosorbent assay (ELISA) method, using an Awareness Microplate Reader Stat Fax 3200 (Awareness Technology Inc., USA). More detailed information about the methods used to measure blood serum proteins is provided by Mohammadi et al.^6^.

### Genotyping and quality control

Genomic DNA was extracted from the EDTA-containing blood samples, using ${DNP}^{TM} Kit$ (CinnaGen Inc, Iran). The samples were sent to Illumina laboratory and genotyped using the Ovine SNP50 BeadChip (Illumina Inc., CA, USA), which detected a total of 48,054 SNPs in the genome. Quality control process was performed using R^28^ and Plink 1.90 beta^29^ software, whereby samples with a GenCall (GC) score < 0.6 and a call rate < 0.99 and variants with minor allele frequencies (MAF) < 0.05, genotype call rates < 0.95 and significant deviation from Hardy-Weinberg equilibrium ($p<{10}^{-6}$) were removed from the analysis.

### Statistical analyses

In the first analysis, the phenotypic records of different serum protein fractions were subjected to a general linear model^4^ as follows:

1 $$ y_{ijkl} = \mu + A_{i} + B_{j} + S_{k} + \beta (EBV_{ijkl} ) + e_{ijkl} $$

In this model, ${y}_{ijkl}$ is an observation, μ, ${A}_{i}$, ${B}_{j}$ and ${S}_{k}$ are overall mean and effects of age (2–3, 4, 5, 6 and + 7 years), birth type (1 or 2) and birth season (winter or spring), respectively, $\beta$ is regression coefficient of the observed parameter on estimate of breeding value for body weight (EBV), as an indicator of genetic potential of body weight and ${e}_{ijkl}$ is residual effects.

EBVs for body weight were obtained using 15859 test-day body weight records of 4402 individuals, collected during 29 years in the studied population. The EBVs were estimated based on Average-Information algorithm of restricted maximum likelihood (AI-REML), using an animal mixed model fitting animal birth year, birth month, birth type, sex and quadratic regression coefficient of body weight on age, as fixed effects and direct additive genetic and permanent environmental and maternal additive genetic effects as random effects.

The general linear and animal mixed models were analyzed using Proc GLM of SAS^30^ and Wombat software^31^, respectively.

Residuals of the general linear model (1), as adjusted records, were considered as pseudo-phenotypes in genome-wide association analysis. The model used for GWAS, fitted random SNP effects and the first five principal components (PCs) as covariates to account the biases due to possible population stratification. The GWAS was carried out as a single-SNP regression and the SNPs were fitted separately. The genome-wide association p-values were adjusted by Bonferroni adjustment method. The Plink 1.90 beta software^29^ was used for genome-wide association analysis. The qqman package of R^32^ was used to create quantile-quantile (Q-Q) and Manhattan plots. Genomic heritabilities and contributions of the significant SNPs in genetic variation of the studied traits were estimated, based on AI-REML algorithm, using GCTA software^33^.

### Gene annotation

Possible candidate genes, located within 50 kbp distances from the detected significant SNPs, based on Bonferonni adjusted p-values, were identified based on SNPchiMp V.3 ovine SNPs genome map^34^, using BioMart tool of Ensembl database (www.ensembl.org). The published QTLs around the significant SNPs were also searched using Animal QTL database (www.animalgenome.org/QTLdb/sheep).

### Approval for animal experiments

The experimental protocols were approved by the Biomedical Ethics Committee of Bu-Ali Sina University. All methods were carried out in accordance with relevant guidelines and regulations. The authors also complied with the ARRIVE (Animal Research: Reporting of In Vivo Experiments) guidelines.

## Results

### Descriptive statistics

Averages ± standard deviations of total protein, albumin, globulins, α1 globulin, α2 globulin, β globulin, γ globulin and IgG, were 5.82 ± 0.97, 2.35 ± 0.51, 3.47 ± 0.82, 0.06 ± 0.05, 0.42 ± 0.17, 0.15 ± 0.14, 2.82 ± 0.76 and 1.56 ± 0.41 g/dL, respectively. Average ± standard deviation of albumin/globulin ratio was 0.69 ± 0.22.

### Quality control

In quality control, two samples had call rates lower than 0.99 (actually < 0.7) and thus were removed from the analysis. A total of 4931 SNPs with MAF < 0.05, 1283 SNPs with genotype call rates < 0.95 and one SNP with significant Hard–Weinberg disequilibrium were also withdrawn from the analysis. As the result of quality control, a total of 94 samples and 41839 SNPs were used for the final analysis.

### Estimates of genomic heritability

Estimates of genomic heritabilities ± SE were 0.000 ± 0.296 for total protein, 0.000 ± 0.299 for albumin, 0.868 ± 0.262 for globulins, 0.227 ± 0.313 for albumin/globulin ratio, 0.264 ± 0.337 for α1 globulin, 0.000 ± 0.288 for α2 globulin, 0.000 ± 0.306 for β globulin, 0.831 ± 0.364 for γ globulin and 0.000 ± 0.266 for IgG.

### Genome-wide association analysis

**Figure 1:**
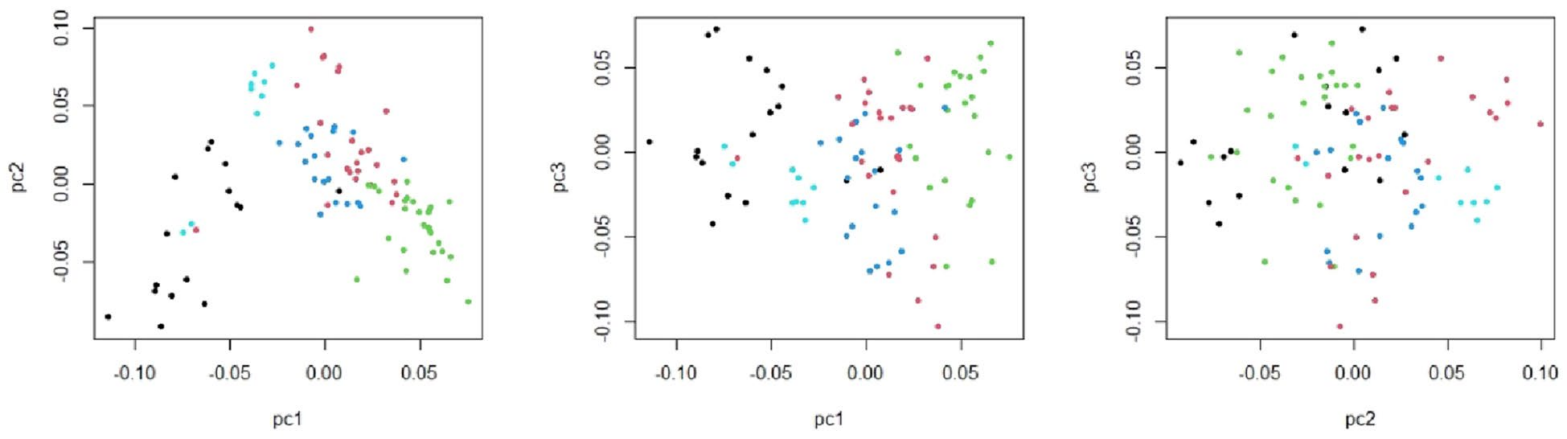
Multi-dimensional scaling (MDS) plots of the genotyping data, based on the first three PCs. Each dot is an animal and different dot colours represent clustering solutions.

Multi-dimensional scaling (MDS) plots of the genotyping data, based on the first three PCs did not show any obvious classification of the sampled animals. However, a slight stratification was observed based on the first two PCs. Whereby a few animals, as the first cluster (black dots) had a slight distance from the others (Fig. 1). This slight stratification is probably due to interfamily differences and import of rams from other populations.

**Figure 2:**
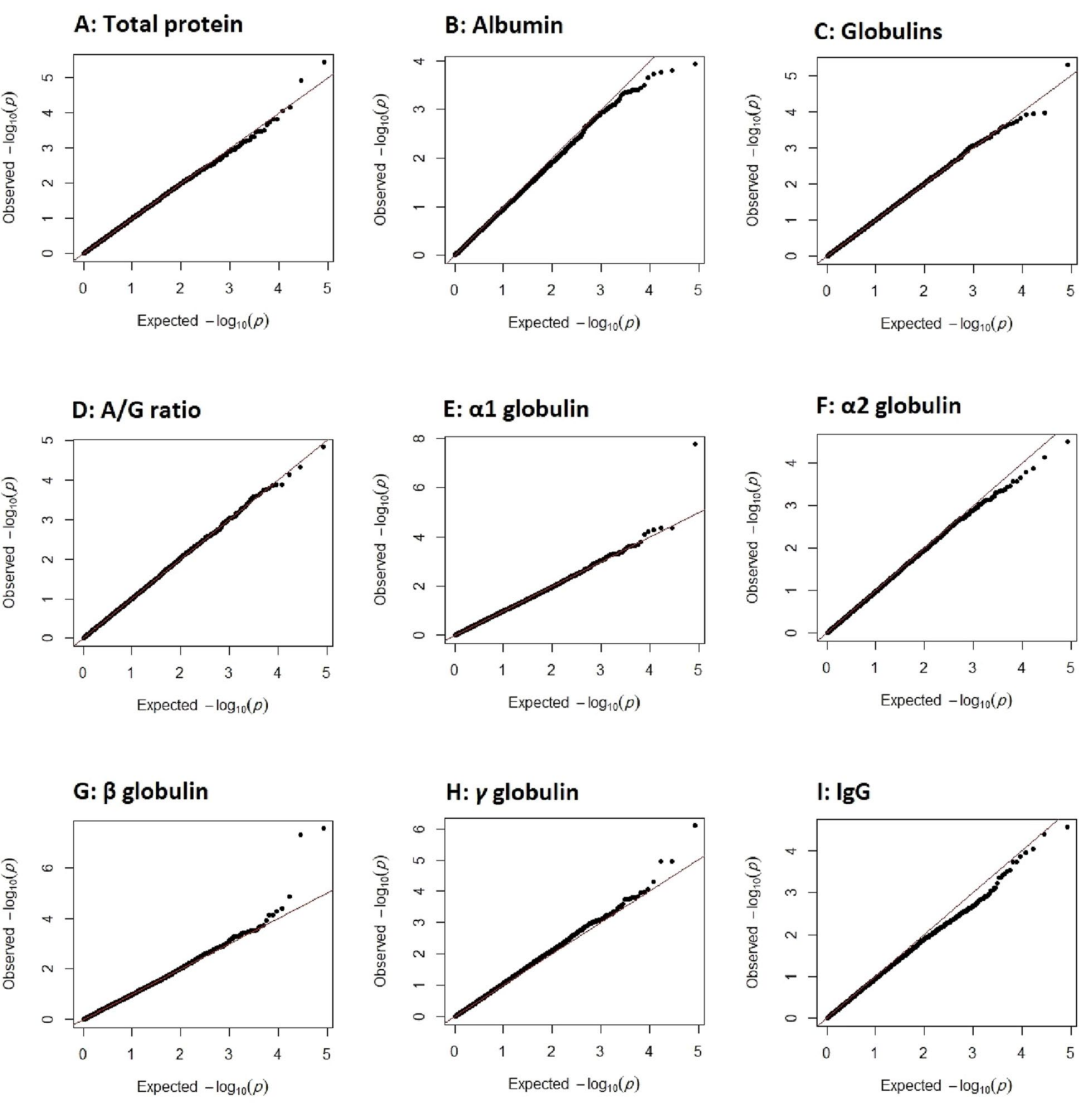
Quantile–quantile (Q–Q) plots for total protein (**A**), albumin (**B**), globulins (**C**), albumin/globulin ratio (**D**), α1 globulin (**E**), α2 globulin (**F**), β globulin (**G**), γ globulin (**H**) and IgG (I). The dots are GWAS − log10 (*p* values) and the line represents the expected values for no association.

Estimates of genomic inflation factor (λ) in the association analysis for total protein, albumin, globulins, albumin/globulins ratio, α1 globulin, α2 globulin, β globulin, γ globulin and IgG were 1.01, 1.00, 1.02, 1.00, 1.00, 1.00, 1.03, 1.07 and 1.00, respectively. Q–Q plots of GWAS −log10 (*p* values) for the studied traits are presented in the Fig. 2.

**Figure 3:**
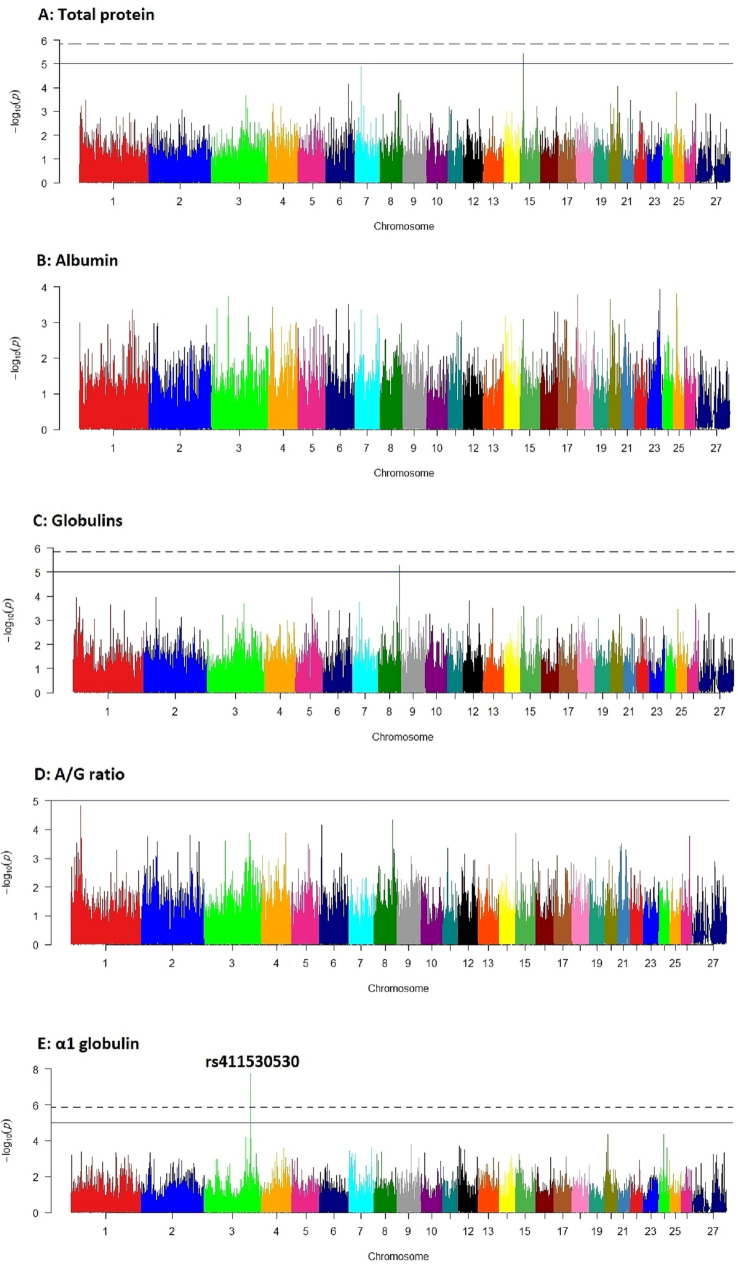

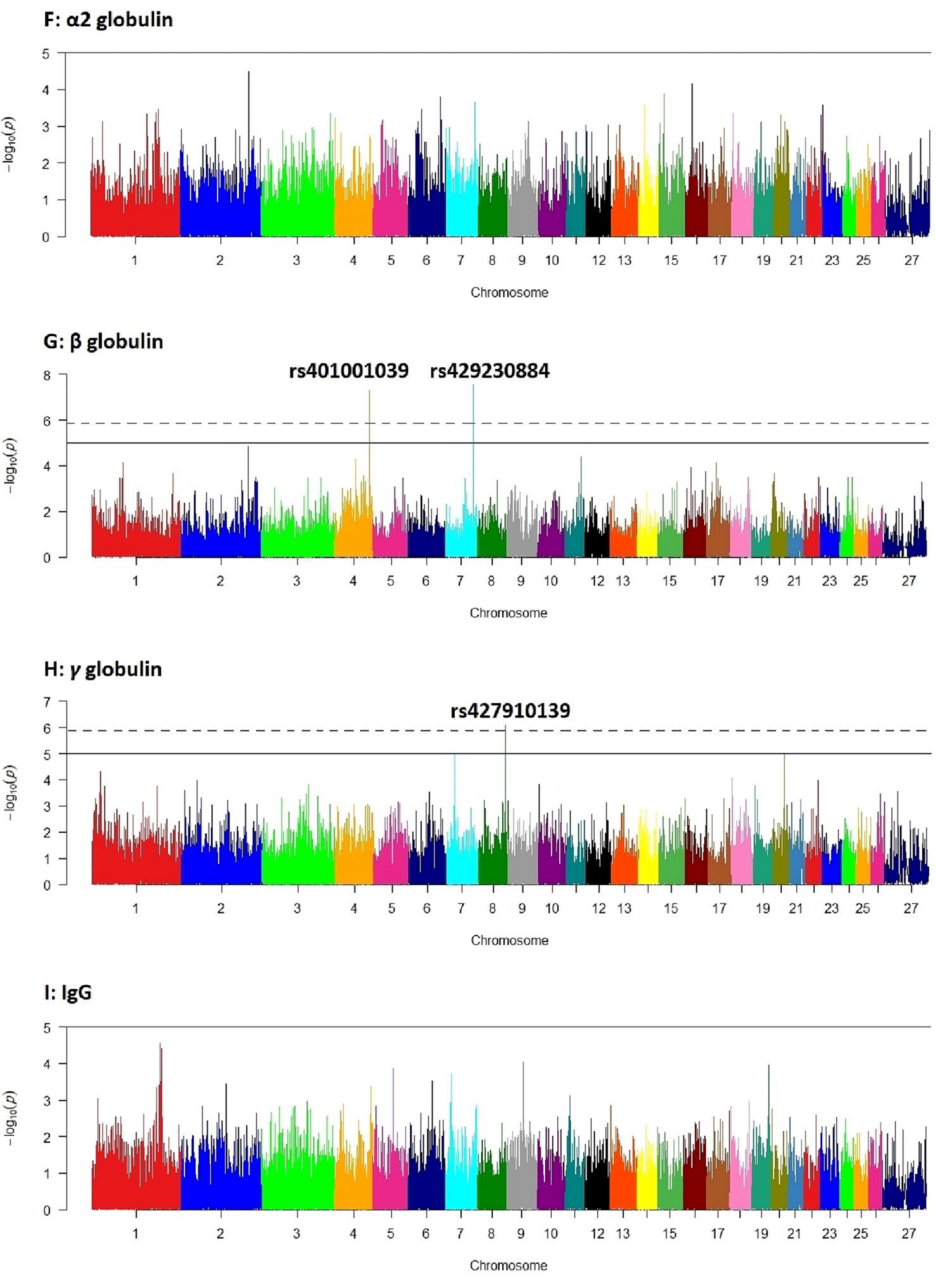
Manhattan plots of genome-wide − log10 (*p* values) for the studied traits. Solid and dashed horizontal lines show genome-wide *p* values of ${10}^{-5}$ and $1.19\times {10}^{-6}$ (Bonferroni adjusted *p* value = 0.05), respectively; A: total protein, B: albumin, C: globulins, D: albumin/globulin ratio, E: α1 globulin, F: α2 globulin, G: β globulin, H: γ globulin and I: IgG.

In genome-wide association analysis, a total of six SNPs had genome-wide *p* values < ${10}^{-5}$. However, based on Bonferroni adjusted *p* values, four SNPs, including one SNP on chromosome 3 (rs411530530), two SNPs on chromosomes 7 (rs429230884) and 4 (rs401001039), and one SNP on chromosome 8 (rs427910139) had significant associations (*p* < 0.05) with α1, β and γ globulins, respectively (Table 1). Other SNPs did not show any significant association with the studied traits. Manhattan plots of GWAS − log10 (*p* values) for the studied traits are presented in the Fig. 3.

**Table 1:** Significant SNPs detected in genome-wide association analysis for the studied traits.

Protein fraction	SNP	Chr.	FS Alleles	Location (bp)	G *p* value	B *p* value
α1	rs411530530	3	T/C	181680527	1.761e–08	0.0007
β	rs429230884	7	A/G	86283700	2.858e–08	0.0012
β	rs401001039	4	A/G	107694115	5.112e–08	0.0021
γ	rs427910139	8	A/G	82987574	8.105e–07	0.0339
TP	rs424180409	15	A/G	14720397	3.671e–06	0.1536
Glo	rs427910139	8	A/G	82987574	5.125e–06	0.2144

α1, β, γ, TP and Glo stand for α1, β and γ globulins, total protein and globulins, respectively; Chr. is chromosome number; FS alleles are forward strand alleles; G *p* value and B *p* value are genome-wide and Bonferroni-adjusted *p* values, respectively; GVE is genetic variance explained by the SNP.

### Genes and QTLs annotation

**Figure 4:**
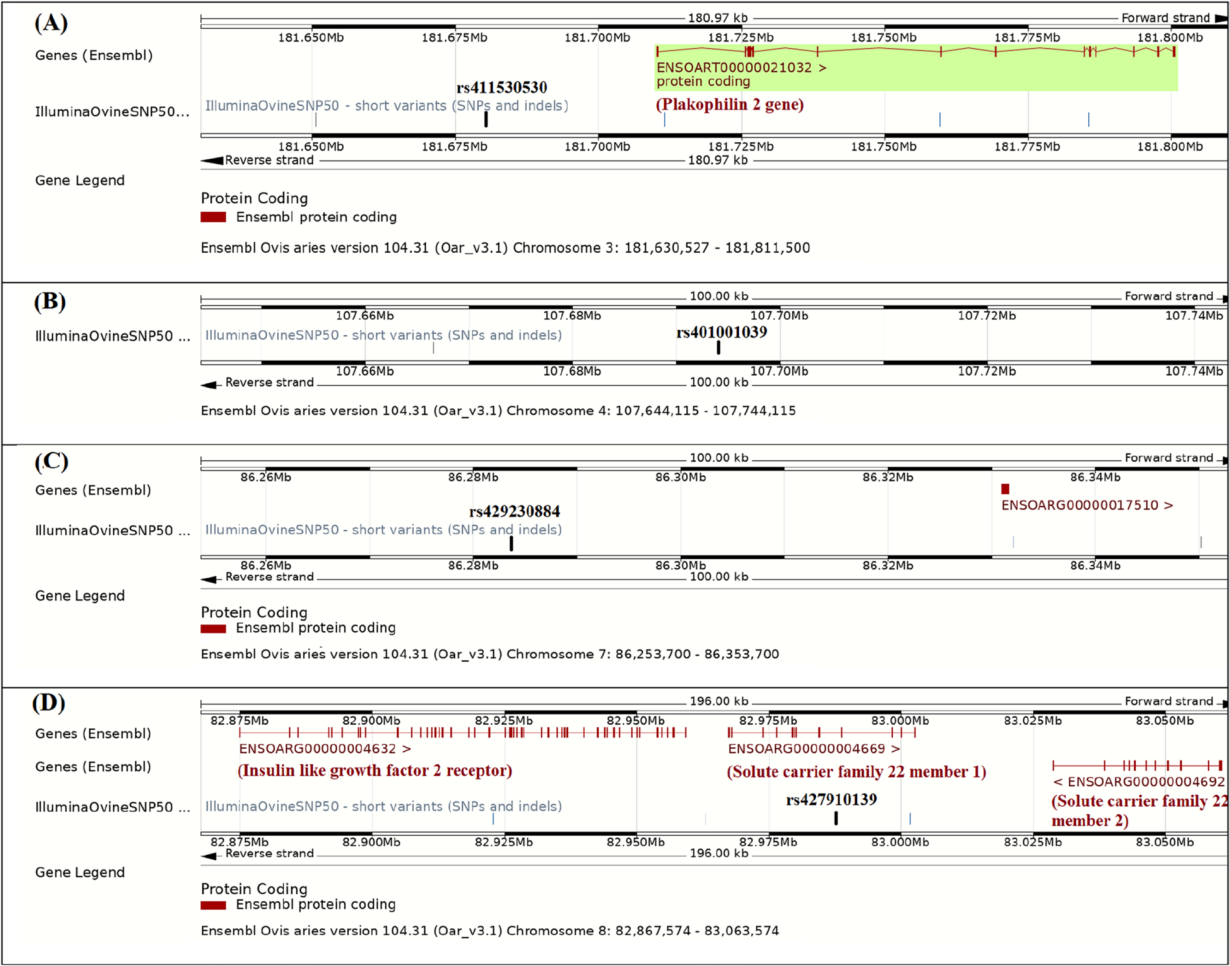
The genes found within 50 kbp distances from the significant SNPs on chromosomes 3 (**A**), 4 (**B**), 7 (**C**) and 8 (**D**), based on BioMart tool of Ensembl (www.ensembl.org).

Based on BioMart tool of Ensembl database (www.ensembl.org), a total of five genes were found within 50 kbp distances from the significant SNPs. The genes found were plakophilin 2 (PKP2) gene on chromosome 3, ENSOARG00000017510 on chromosome 7, and three genes, including insulin like growth factor 2 receptor (IGF2R), solute carrier family 22 members 1 and 2 (SLC22A1 and SLC22A2, respectively) on chromosome 8. No gene was found within 50 kbp intervals from the significant SNP on chromosome 4 (Table 2). The genes surrounding the significant SNPs on chromosomes 3, 4, 7 and 8 are illustrated in Fig. 4. Based on the Animal QTL database (Animal QTLdb), no QTL associated with the studied blood serum proteins was found around the significant SNPs. However, some QTLs associated with immunoglobins A (IgA), E (IgE) and G (IgG) were found in the Animal QTL database.

**Table 2:** The genes found in 50 kbp distances from the detected significant SNPs in GWAS, based on BioMart tool of Ensembl database (www.ensembl.org).

SNP	Chr	Location (bp)	Gene	Strand	Gene start (bp)	Gene end (bp)	Distance
rs411530530	3	181680527	PKP2	F	181710282	181800739	− 29755
rs401001039	4	107694115	–	F	–	–	–
rs429230884	7	86283700	ENSOARG00000017510	F	86330934	86331638	− 47234
rs427910139	8	82987574	IGF2R	F	82874886	82959397	28177
			SLC22A1	F	82967383	83002611	Within
			SLC22A2	R	83028720	83060568	− 41146

PKP2: Plakophilin 2; IGF2R: Insulin like growth factor 2 receptor; SLC22A1: Solute carrier family 22 member 1; SLC22A2: Solute carrier family 22 member 2; F and R: forward and reverse strands, respectively; Negative and positive distances indicate the SNPs before and after the genes, respectively.

## Discussion

Total protein and globulins, albumin, albumin to globulins ratio and γ globulin averages were in the range, reported for different populations, such as Merino sheep^13,35^, Karakul and Tzurcana ewes^10,13^, Balami ewes^14^, Lori-Bakhtiari and Mehraban sheep^36^ and Santa Inês ewes^37^. However, averages of α1, α2 and β globulins levels were to some extent lower than those reported in literature^9,13,36^. The observed differences are probably due to different environmental, physiological, health, age and genetic conditions of the studied populations. For example, it has been found that sick animals may have higher levels of α and β globulins^9^.

In the present study, moderate to high genomic heritabilities were estimated for total globulins (0.868), albumin/globulin ratio (0.227), α1 globulin (0.264) and γ globulin (0.831), which indicates considerable genetic effects on these protein fractions and probably their potential use as biomarkers for genetic selection. Other fractions, with negligible heritabilities, including total protein, albumin, α2 globulin, β globulin and IgG are likely proper guides to animal management^38^. The present study is probably the first published attempt to estimate genomic heritabilities of the blood protein levels. However, based on high standard errors of the heritability estimates, which was due to limited number of the sampled animals, more studies are still needed to clarify exact genetic bases of blood serum protein variations.

The significant SNPs detected on chromosomes 3, 4, 7 and 8 (Table 1) were not located near to the reported QTLs associated to blood serum proteins. However, a total of 32 QTLs for IgA on chromosomes 1, 2, 3, 4, 6, 9, 10, 11, 12, 13, 15, 16, 17, 20, 21, 22 and 23, one QTL for IgE on chromosome 23 and four QTLs for IgG on chromosomes 5, 9 and 23 were found in Animal QTL database (www.animalgenome.org/QTLdb/sheep). Based on Animal QTLdb, the reported QTLs for IgA on OAR3 and OAR4 are located on 138.6–150.3 Mbp of OAR3^39^, 155.9–156.0 Mbp of OAR3^40^, 200.2 and 209.7 Mbp of OAR3^41^, 54.0–58.0 of OAR4^42^, and 76.4 and 82.5 Mbp of OAR4^41^, which are different from the detected SNPs on 181.7 Mbp of OAR3 and 107.7 Mbp of OAR4 in the present study (Table 1). No QTL was found for serum total protein, albumin, globulins, α1, α2, β and γ globulins in Animal QTLdb for sheep. However, despite QTLs, a total of five genes, including *PKP2*, ENSOARG00000017510, *IGF2R*, *SLC22A1* and *SLC22A2* were found in 50 kbp distances from the significant SNPs on chromosomes 3, 7 and 8 (Table 2).

A few genome-wide association studies have been conducted on blood serum proteins. A GWAS on Korean population revealed six loci, including *TNFRSF13B*, *FADS1*, *GALNT2*, *IRF4*, *HLA*-*DBP1* and *SLC31A1*, associated with albumin/globulin ratio^20^. In a GWAS on Japanese human population, based on two significant SNPs for total protein and one significant SNP for albumin, five genes, including *TNFRSF13B*, *RPL13A*, *RPS11*, *FCGRT* and *RCN3* and one gene, *GCKR* were suggested as candidate genes for total protein and albumin levels, respectively^22^. Associations of the *TNFRSF13B* and *GCKR* genes with total protein and albumin levels were also confirmed in another GWAS on Japanese people^24^. In a GWAS meta-analysis on east-Asian human population, one SNP, located within the *RRPS11* gene, was significantly associated with blood plasma albumin level^23^. Almost different candidate genes for blood protein fractions were found in the present and previous GWASs, which is probably due to population-specific associations^22^ and different genetic architectures of the studied populations and individuals^43^.

The plakophilin 2 gene (*PKP2*), located on OAR3, encodes the plakophilin 2 protein, which is mainly found in myocardium cells the heart wall. This protein is a found in desmosome structures, a component of intercellular adhesive junction^44,45^. The compromised junctional integrity probably contributes to disease pathophysiology^46^. Although PKP2 serves as a critical scaffold for protein kinase Cα (PKCα). Thus, more global functions in cellular homeostasis are expected for PKP2. It has been found that *PKP2* knockdown would result in increased PKC substrate phosphorylation, and this association is probably the reason for pathogenesis of congenital defects due to PKP2 deficiency^46^. In several studies, the *PKP2* gene was associated with canine atopic dermatitis, a chronic inflammatory skin disease^47,48^. It has been found that the *PKP2* impacts the β-catenin activity, a main participant in canonical Wnt signaling and associates with *SOX2* and *SOX9* expressions, as Wnt target genes, which suggests a signaling role of plakophilin 2 by regulation of Wnt signaling pathway^49^. On the other hand, it is demonstrated that the Wnt signaling is essential in pathogenesis of some diseases^50^. Thus, the *PKP2* gene could be considered as a candidate gene for blood serum proteins.

The ENSOARG00000017510 gene, is located on 86.3 Mbp of OAR7, in a 47.2 kbp distance from the SNP rs429230884 (Table 2). Based on the Ensembl database (www.ensembl.org), the ENSOARG00000017510 is a protein-coding gene, which encodes an integral component of membrane. No evidence for phenotypic association of this gene with diseases or other traits was found in literature. It seems that more studies are needed to understand the molecular, cellular and biological functions of this gene.

The insulin like growth factor 2 receptor (IGF2R) is locates on OAR8 and encodes a highly conserved transmembrane glycoprotein receptor which regulates the insulin-like growth factor 2 (IGF2) level and this function is necessary for embryonic development in mammals^51^. However, there are some evidences for IGF2R functions in immunity such as regulation of HIV infection and chemokine expression^52^, overexpression of IGF2R in osteosarcoma cells^53^ and increase of regulatory T cell functions in reducing of other effector T cells activities and suppression of food allergic effects on intestinal inflammation^54^. Moreover, it has been found that some viral infections are associated with IGF system ^52^. Therefore, the *IGF2R* is probably a candidate gene for blood serum proteins and immune system activity.

The significant SNP on OAR8 (rs427910139) is located within and in 41 kbp distance from the solute carrier family 22 members 1 (*SLC22A1*) and 2 (*SLC22A2*), respectively (Fig. 4, part D). The *SLC22A1* and *SLC22A2* encode organic cation transporters, with crucial roles in elimination of endogenous organic cations, drugs and toxins^55^. Associations of SLC22 members 1–3 with drug disposition, response and generally pharmacodynamics are well known^56,57^. There are several evidences for associations of *SLC22A1* and *SLC22A2* with diseases. For example, the *SLC22A2* is associated with hypertension^58^ and *SLC22A1* and *SLC22A2*, both contribute in disposition pathways for fluoroquinolone antimicrobials^59^. In a GWAS on Korean human population, another member of the solute carrier families (*SLC31A1*) was significantly associated with blood serum albumin/globulin ratio^20^. Therefore, both *SLC22A1* and *SLC22A2* could be considered as candidate genes for blood serum protein profile and probably resistance to diseases.

## Conclusion

The QTLs on 181.7 Mbp of OAR3, 107.7 Mbp of OAR4, 86.3 Mbp of OAR7 and 83.0 Mbp of OAR8 in the present study are probably the first QTLs reported for α1, β and γ globulins. Moreover, the *PKP2*, *IGF2R*, *SLC22A1* and *SLC22A2* genes could be considered as candidate genes for blood serum proteins. Moderate to high genomic heritabilities were estimated for total globulins (0.868), albumin/globulin ratio (0.227), α1 globulin (0.264) and γ globulin (0.831). This study showed considerable genetic variation in blood serum protein profile, especially total and gamma globulins. This study is probably the first GWAS on blood serum protein profile in animals. However, more studies with larger sample sizes and use of high-density SNP chips would probably result in detection of more genomic regions associated with blood serum protein profile in sheep.
